# Glycans as Modulators for the Formation and Functional Properties of Neutrophil Extracellular Traps: Used by the Forces of Good and Evil

**DOI:** 10.3389/fimmu.2019.00959

**Published:** 2019-05-07

**Authors:** Kim F. Bornhöfft, Sebastian P. Galuska

**Affiliations:** Institute of Reproductive Biology, Leibniz Institute for Farm Animal Biology, Dummerstorf, Germany

**Keywords:** NETosis, Siglecs, polysialic acid, histones, glycosaminoglycans

## Abstract

A very common mechanism to trap pathogens is the release of DNA. Like flies in a spider's web, pathogens are enclosed in a sticky chromatin meshwork. Interestingly, plants already use this mechanism to catch bacteria. In mammals, especially neutrophils release their DNA to prevent an invasion of bacteria. These neutrophil extracellular traps (NETs) are equipped with antimicrobial molecules, including, for instance, histones, antimicrobial peptides, lactoferrin, and neutrophil elastase. Thus, in a defined area, pathogens and toxic molecules are directly adjacent. However, several of these antimicrobial substances are also cytotoxic for endogenous cells. It is, therefore, not surprising that distinct control mechanisms exist to prevent an exaggerated NETosis. Nevertheless, despite these endogenous control instruments, an extraordinary NET release is characteristic for several pathologies. Consequently, NETs are a novel target for developing therapeutic strategies. In this review, we summarize the roles of glycans in the biology of NETs; on the one hand, we focus on the glycan-dependent strategies of endogenous cells to control NET formation or to inactivate its cytotoxic effects, and, on the other hand, the “sweet” tricks of pathogens to inhibit the release of NETs or to prevent NET-mediated killing mechanisms are examined. Understanding both, the forces of good and evil, allows the development of novel glycan-based approaches to combat the harmful side of NETs during distinct pathologies.

## Introduction

Neutrophil granulocytes possess a panel of different mechanisms to combat invading pathogens. As first line of defense neutrophils can combat pathogens by phagocytosing them, by releasing antimicrobial peptides or reactive oxygen species (ROS) and by the release of neutrophil extracellular traps (NETs), a process first described in 2004 by Brinkmann et al. (1, 2). NET release can be induced by bacteria, viruses, fungi or non-physiological stimuli, such as ionophores and phorbol-myristate acetate (PMA) (3, 4). It seems to be that different pathways can be used to induce the formation of NET and even more than 13 years after the first description of NETs several mechanisms are controversially discussed and numerous open questions still need to be answered [excellently summarized in Boeltz et al. (5)]. What we know is that NET consists of a meshwork of decondensed DNA fibers, cytotoxic histones, and antimicrobial peptides and has the ability to catch and render invading pathogens harmless (3, 5, 6). Since NET contains several biomolecules, which are also cytotoxic for endogenous cells, in addition to the desired antimicrobial effects, NETs are associated with numerous pathologies (7–16). Therefore, an exaggerated release of NET has to be prevented, leading to the necessity of control mechanisms to regulate the formation of NETs.

Within the last few years, interest in glycans and how they modulate different functions has increased immensely (17–19). Since all of our cells are surrounded by a glycocalyx consisting of highly glycosylated proteins and lipids, it is obvious that glycan-dependent processes occur frequently (19, 20). Indeed, glycans have essential roles within various biological processes, such as cell proliferation, cell differentiation, the development of organs, and within the immune system (19, 21). In the field of immunology, glycans drive diverse mechanisms, ranging from the discrimination of the self and non-self, using for instance sialic acid-binding immunoglobulin-like lectins (Siglecs), to glycosaminoglycan (GAG)-mediated chemokine presentation (22).

GAGs are a number of long, unbranched polysaccharides composed of repeating disaccharide units, whereby, the repeating disaccharides consist of uronic acid or galactose and an amino sugar, which can be additionally modified. Prominent members of the GAG family are: hyaluronic acid, heparin/heparan sulfate, chondroitin sulfate/dermatan sulfate, and keratin sulfate (23). Besides their role in chemokine presentation, GAGs are also involved in several other biological processes, like cell signaling, angiogenesis (24), metastasis, tumor progression (25, 26) and coagulation (27, 28).

In addition to GAGs, sialylated glycans play an important role during immunological events. In mammals, glycans are frequently terminated by these sugar residues (29). Sialic acids are negatively charged and modulate both immunological processes and organ development (30–33). Sialylated structures can be recognized by Siglecs (34–36), which are transmembrane receptors expressed in different cells of vertebrates that can mainly either inhibit or activate the immune response (35, 37–39). Although Siglecs are meant to be a valuable tool to distinguish between self-associated molecular patterns (SAMPs) and pathogen-associated molecular patterns (PAMPs), several pathogens are already known to elude the immune system by mimicking host sialylation and, therefore, masking themselves as SAMPs.

This review is therefore focusing on the role of glycoconjugates of endogenous cells in controlling NET releases and impairing the negative outcome of NETs as well as on the role of glycoconjugates of pathogens that influence NET formation or that directly influence NET release and the biological activity of NETs.

## The Interaction Between Glycans and Neutrophils: a Physiological Control System in Circulation

An impaired NET release and missing NET clearance are, for instance, associated with the formation of a thrombus (7, 40, 41). Not only do the NET fibers serve as a scaffold for the formation of a thrombus. Furthermore, neutrophil elastase is released in the extracellular area and inhibits among others the anticoagulants antithrombin and tissue factor pathway inhibitor. In addition, extracellular histones are known to increase the thrombin generation, causing platelet activation and coagulation (40–43). Therefore, it is not surprising that an impaired NET release within circulation needs to be prevented.

**Figure 1:**
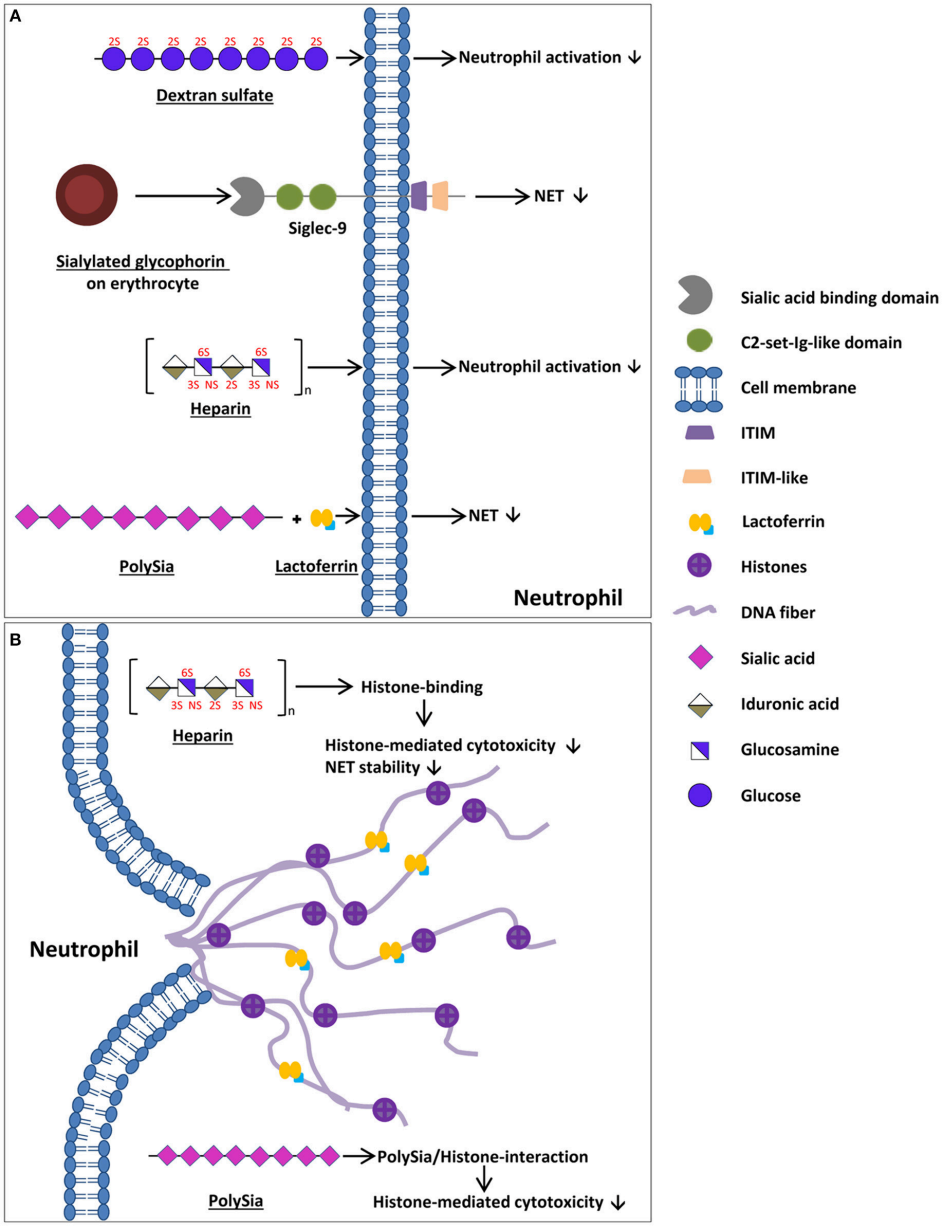
Mechanisms of endogenous cells and naturally occurring components to enhance bacterial entrapment, to decrease impaired NET release, and to decrease the cytotoxic outcome of NETs. **(A)** Natural occurring components preventing NET release/neutrophil degranulation. Glycophorin is a sialoglycoprotein located at the surface of erythrocytes that inhibits NET release/neutrophil activation via sialic acid binding to Siglec-9 within circulation. Furthermore, the GAG heparin as well as dextran sulfate inhibit neutrophil activation in a sulfate-dependent manner. The interaction of lactoferrin with polySia increases the inhibition of NET releases. **(B)** Mechanisms to decrease the cytotoxic outcome of NETs for a body's own cells as well as to increase bacterial entrapment. PolySia as well as GAGs, such as heparin, bind to released histones, reducing histone-mediated cytotoxicity. The cartoons of all polysaccharides show only exemplary parts of these polymers and may differ from the actual structure (e.g., chain length and composition).

Lizcano et al. investigated the reason why isolated neutrophils are more susceptible to undergoing NETosis than neutrophils within circulation (44). They determined that glycophorin A, a sialoglycoprotein that is located on the surface of erythrocytes, is a candidate that might be responsible for this effect. It is able to bind Siglec-9 on the surface of neutrophils, inhibiting neutrophil activation within circulation (Figure 1A). Remarkably, modification of the sialic acid on the surface of the erythrocytes prevents the outlined inhibitory effects (44). Interestingly, also cancer cells, which are characterized by hypersialylation, seem to use Siglec-5 and Siglec-9 to evade entrapment by activated neutrophils [excellently reviewed by Adams et al. (45) and Rodrigues and Macauley (46)].

Besides sialylated glycoconjugates, heparin, a well-known anticoagulant, inhibits neutrophil degranulation and aggregation *in vitro* (47). Furthermore, the influence of low molecular weight heparin, unfractionated heparin, O-desulfated heparin, hyaluronic acid, dextran sulfate, and poly-L-glutamic acid on neutrophil activation was investigated (48). The activation of neutrophils with different stimuli induced the release of neutrophil elastase. However, the application of the different stimuli in combination with low molecular weight heparin as well as dextran sulfate inhibited neutrophil activation and, therefore, the release of neutrophil elastase (Figure 1A) (48, 49). In contrast, the non-sulfated dextran and poly-L-glutamic acid showed no effect on neutrophil activation, leading to the assumption of a sulfate-dependent process. Furthermore, Xu et al. investigated the role of heparan sulfate in the biology of NETs (50). In heparan sulfate uronyl 2-O-sulgotransferase deficient mice less NET is formed after stimulation with group B streptococcus (GBS). Remarkably, when NET was treated with heparan lyase, its antimicrobial activity decreased (50). Thus, the formation and the activity of NETs seem to be modulated by heparan sulfate.

Besides heparan sulfate, other glycans are known to influence the biological activity of NETs. In this context, Brown et al. focused on neutrophil elastase and neutrophil-induced human bronchial epithelia cell detachment (48). Whilst hyaluronic acid had no effect, low-molecular-weight heparin, unfractionated heparin, O-desulfated heparin, and dextran sulfate significantly inhibited the neutrophil elastase-induced detachment (48). Furthermore, Fuchs et al. published that the treatment of NETs with heparin destroys their scaffold and prevents the formation of a thrombus (Figure 1B). Heparin has a high-charge-dependent affinity to histones (51, 52) and is able to release histones from chromatin fibers, therefore, destabilizing NETs (Figure 1B) (40). Since histones that are released during NETs are able to damage negatively charged cell membranes, histones are often described as antimicrobial peptides (AMPs) that are released during NETs alongside with other antimicrobial biomolecules like lactoferrin and neutrophil elastase (1, 6, 53–56). Unfortunately, these properties of all histones (H1, H2A, H2B, H3, and H4) are toxic not only for pathogens but also for endogenous cells (57–60). Within the plasma, the cytotoxicity of histones is reduced by the inter-alpha-inhibitor-protein (IAIP) associated with high molecular weight hyaluronic acid and chondroitin sulfate. IAIP as well as high molecular weight hyaluronic acid and chondroitin sulfate bind recombinant histone H4, contributing to reduced histone mediated cytotoxicity (Figure 1B) (61).

In addition to GAGs, such as hepain (52), another linear carbohydrate, polysialic acid (polySia), is a naturally occurring inhibitor of the cytotoxic effects of histones (59, 62, 63). Remarkably, polySia was detected in the plasma of different species, from fish to humankind, and may represent a natural buffer system for the inactivation of the cytotoxicity of extracellular histones in blood (Figure 1B) (64). PolySia influences histone-mediated cytotoxicity in a concentration as well as in a chain-length-dependent manner (65, 66). In line with that, polysialylated nanoparticles and *in vitro* polysialylated cervical mucins represent tools to counteract histone-mediated cytotoxicity during an exaggerated NET formation (65, 66). Interestingly, quite recently, Kühnle et al. (67, 68) published that polySia interacts with lactoferrin. Lactoferrin is known to inhibit a NET release by forming a “lactoferrin-shell” around the activated neutrophils (69). *In vitro* experiments suggested that the efficiency of lactoferrin in preventing the release of NETs was enhanced in the presence of polySia (67).

Thus, the presented examples show endogenous glycan-dependent ways to control the release of NETs in addition to decreasing their damaging effects, indicating, once more, the widespread function of glycosylation within the field of immunology. However, it has to be considered that a medal always has two sides. Some pathogens exploit the previously described mechanisms for their own purposes, as described in the next chapter.

## The Glycosylation of Pathogens: a Powerful Tool to Escape NETs

### Bacteria

**Figure 2:**
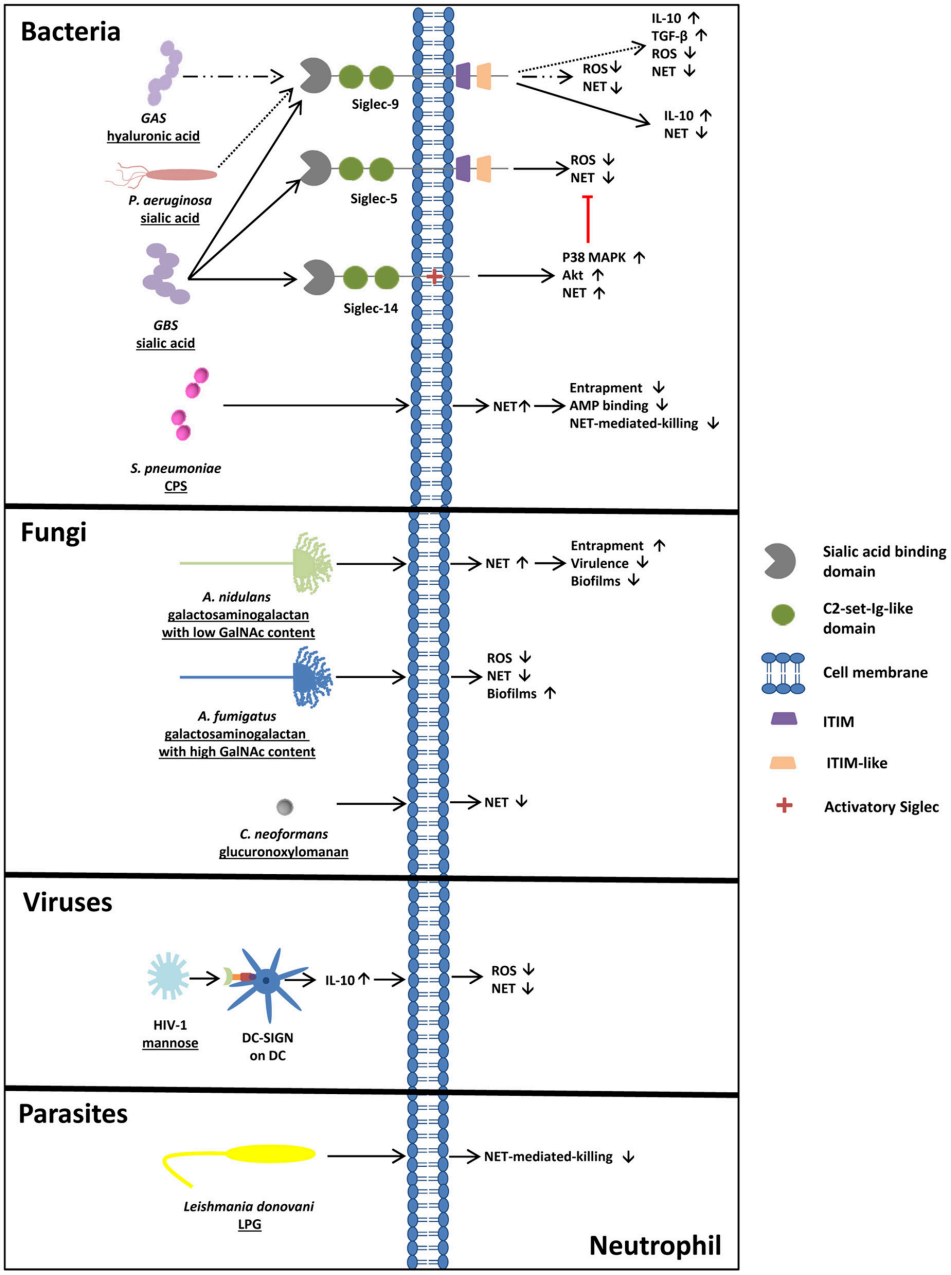
Glycosylation of pathogens- a powerful tool to circumvent NET-mediated entrapment and killing. Bacteria circumventing NET release or NET-mediated killing. *S. pneumoniae* induces NET release, but NET-mediated killing and entrapment is bypassed due to the capsular polysaccharide (CPS). Pathogens like GBS, GAS, and *P. aeruginosa* exploit Siglecs for their own purposes. Via sialic/hyaluronic acid binding to Siglecs, IL-10 as well as TGF-β production are upregulated and NET release is inhibited. Also fungi circumvent NET release/ NET entrapment by glycans. Compared to the less pathogenic *A. nidulans*, surrounded by galactosaminogalactan with a low amount of GalNAc, *A. fumigatus*, surrounded by galactosaminogalactan with a high GalNAc content, inhibits NET release and ROS production and forms more adherent biofilms, explaining the differences in virulence of these two fungi strains. In addition, the capsular strain of *C. neoformans* inhibits the release of NETs due to its glucuronoxylomannan coating, whereas acapsular strains of *C. neoformans* induce NETosis. In addition, sialylated viruses like HIV-1 prevent ROS-dependent NET release by DC-SIGN engagement and parasites, like *Leishmania donovani* circumvent NET mediated killing by lipopeptidoglycan (LPG) on its surface.

During their evolution, several pathogens have “learned” to use carbohydrate-dependent mechanisms to modulate the immune system. For instance, distinct bacteria strains target Siglecs to circumvent the release of NETs by neutrophils. *Pseudomonas aeruginosa (P. aeruginosa)*, for example, can use sialic acids from its hosts to decorate their glycoconjugates with sialic acids. On the surface of *P. aeruginosa, N*-acetylneuraminic acid *(*Neu5Ac), *N*-glycolylneuraminic acid (Neu5Gc), and 9-O-acetyl-*N*-acetylneuraminic acid (Neu5,9Ac2) have been found, enabling *P. aeruginosa* to inhibit neutrophil activation via the activation of Siglec-9 (70). According to Khatua et al. *P. aeruginosa* directly binds to the neutrophil via Siglec-9, stimulating the production of cytokine IL-10 and TGF-β (71). The generation of ROS is inhibited, and a decrease of the release of neutrophil elastase is detected. Since ROS production can be an initial step of NETosis, it is not surprising that the release of NETs is inhibited by the binding partners of Siglec-9 on the surface of *P. aeruginosa* (Figure 2) (72, 73).

However, *P. aeruginosa* is not the only pathogen using Siglec-9 to circumvent the activation of neutrophils. Carlin et al. (74) also showed that GBS is able to inhibit neutrophil activation through Siglec-9. GBS eluded the human immune system, causing invasive infections in human newborns by hosting the common terminus of human glycoproteins, Siaα(2,3)Galβ(1,4)GlcNAc, on their capsular polysaccharide. Here, the sialic acid binding of GBS to neutrophil Siglec-9 also initiated the production of the NET-suppressive cytokine IL-10 (74).

In addition to these findings, glycans of GBS interact with Siglec-5 and Siglec-14 (75). These Siglecs are an example of an antagonistic interplay between Siglecs. The sialic acid binding of Siglec-14 counteracts the pathogen-induced suppression of neutrophil activation (Figure 2). Intriguingly, the absence of Siglec-14 due to Siglec-14 null-polymorphism in humans leads to the increased susceptibility of neutrophils to GBS. The relevance of this pathogen-induced inhibition of neutrophil activation becomes apparent through the discovery of the Siglec-5 and Siglec-14 expressions on amniotic epithelium (75). These epithelial cells are the site of the initial contact area of the fetus and the pathogens. Ali et al. suggested that Siglec-14 null-polymorphism might relate to the risk of prematurity during GBS invasion.

In 2016, a surprising discovery was made by Secundino et al. (76). While investigating GBS and its capability to bind and activate Siglec-9 via its sialylated glycans, they observed that Siglec-9 also bound high molecular weight hyaluronan, consisting of repeating disaccharide units of *N*-acetylglucosamine (GlcNAc) and glucuronic acid (GlcA), with alternating β1,4- and β1,3-linkages. Intriguingly, they detected a new, specific binding site, apart from the V-set Ig-like domain. Since the capsular polysaccharide of group A streptococcus (GAS) contains high-molecular-weight hyaluronan units, NETs' formation and oxidative bursts were prevented (Figure 2) (76). Remarkably, a single inhibitory Siglec recognizes two different glycan motifs as SAMPs, leading to the suppression of neutrophil activation.

Although GBS seems to be the best studied pathogen regarding sialic acids and NETs inhibition, several more pathogens, like *Campylobacter jejuni, Neisseria gonorrhoeae*, and *Escherichia coli* K1, are also able to synthesize sialylated glycans, leading to the assumption that comparable strategies are also used here to escape NETs (70, 77–79). Since polySia is also able to modulate NET formation and the activity of NETs, it seems likely that polySia-positive bacteria, like *Escherichia coli* K1 and distinct *Neisseria meningitides* strains, are able to trigger polySia-dependent mechanisms (80, 81). However, until now, no study has examined the impact of polySia during their invasion in the context of NETs.

Interestingly, obstructing NET release is not the only tool of bacteria, to elude the immune system. Several pathogens are able to circumvent NET-mediated killing. *Streptococcus pneumoniae* (*S. pneumoniae*) for instance, which is one of the major causes of mortality and morbidity, circumvents NET-mediated killing through its polysaccharide capsule (82). Encapsulated *S. pneumoniae* strains show significantly reduced trapping by NETs compared to non-capsulated strains (Figure 2). Furthermore, *S. pneumoniae* contain positively charged lipoteichoic acid on their surface, which increases the electrochemical repulsion of antimicrobial peptides (82).

### Fungi

Moreover, it is not only bacteria that circumvent NET-mediated entrapment and killing. In their research, Rocha et al. (83) detected that the fungus *Cryptococcus neoformans* (*C. neoformans*) is surrounded by capsular polysaccharides containing glucuronoxylomannan. The wild type of strain inhibits the release of NETs, whereas acapsular mutants or mutants surrounded by glucuronoxylomannogalactan induce NETosis (Figure 2) (83).

Furthermore, *Aspergillus fumigatus* (*A. fumigatus*), which accounts up for around 80% of all invasive *Aspergillus* infections, shows resistance against NET-induced damage due to the production of cell-wall-associated galactosaminogalactan and a secreted form of galactosaminogalactan. Galactosaminogalactan consists of galactose and N-acetylgalactosamine (GalNAc) residues. Since galactosaminogalactans play a certain role in host-pathogen interactions, as they are required for biofilm formation, a galactosaminogalactan-deficient mutant of *A. fumigatus* exhibited reduced virulence (Figure 2) (84–88).

Interestingly, *Aspergillus nidulans (A. nidulans)*, a strain producing galactosaminogalactans with a lower content of GalNAc residues in comparison to *A. fumigatus*, was found to be less pathogenic, formed less-adherent biofilms, and was, therefore, more susceptible to NET-induced damage. Since *A. nidulans* is only known to induce pathologies in patients with chronic granulomatous disease (CGD), characterized by an impaired NADPH oxidase complex, further investigations concerning the influence of NADPH oxidase revealed that cell-wall-bound galactosaminogalactans in *A. fumigatus* enhance resistance against NADPH-oxidase-dependent neutrophil extracellular damage. This might explain the increased virulence of *A. nidulans* in CGD patients (88).

### Viruses

Interestingly, also viruses are able to evade immune control mechanisms. The HIV-1 virus, for instance, counteracts NET formation by engaging the C-type lectin DC-SIGN (CD209) on dendritic cells via its envelope glycoprotein containing more high mannose than complex N-glycan structures. The binding induces the production of IL-10, contributing to the inhibition of ROS-dependent NET release upon TLR7 and TLR8 engagement (89) (Figure 2). This study let suggest that also glycans of other viruses can target DC-SIGN, such as Ebola virus, the Japanese encephalitis virus and of the hepatitis C virus, that may also influence in an indirect way the formation of NET (90–92).

### Parasites

*Leishmania donovani*, a protozoan parasite, contains lipopeptidoglycan on its surface and mutants lacking lipopeptidoglycan show less survival in NET in comparison to the wild type strain (Figure 2) (93). In addition, virulent strains of *Leishmania donovani* can contain high amount of sialylated glycans representing binding partners for Siglec-5 leading to an inhibition of ROS production in macrophages (94). Comparable mechanism of these *Leishmania donovani* glycans, which are terminated with α2,3- and α2,6-linked sialic acid residues, may also take place on neutrophils and might also be used by other sialic acid positive parasites.

## Conclusion

This review gives a short summary concerning the impact of glycans to modulate the formation and activity of NET describing glycan dependent mechanisms of endogenous cells to prevent the activation of neutrophils or to inactivate cytotoxic molecules of NET, which are, however, also used as an escape strategy by distinct pathogens. All of the outlined examples show that glycans play a key role in the biology of NET and that they have great potential as a therapeutic tool in NETs-associated pathologies.

## Author Contributions

All authors listed have made a substantial, direct and intellectual contribution to the work, and approved it for publication.

### Conflict of Interest Statement

The authors declare that the research was conducted in the absence of any commercial or financial relationships that could be construed as a potential conflict of interest.
